# Treatment Persistence of Paliperidone Palmitate 3‐Month in Patients With Schizophrenia: A Japan Medical Data Center Claims Database Analysis

**DOI:** 10.1002/npr2.70019

**Published:** 2025-05-12

**Authors:** Akihide Wakamatsu, Madoka Chinen, Hiroshi Horio, Chih‐Lin Chiang, Natsuko Tokushige, Yosuke Saga

**Affiliations:** ^1^ Medical Affairs Division Janssen Pharmaceutical K.K. Tokyo Japan; ^2^ Medical Affairs Janssen Pharmaceutical of Johnson & Johnson Taipei City Taiwan

**Keywords:** database, paliperidone palmitate, persistence rate, schizophrenia, treatment switching

## Abstract

**Aim:**

To examine treatment persistence rates of paliperidone palmitate 3‐month (PP3M) for schizophrenia in Japan because evidence in real‐world settings is limited.

**Methods:**

A retrospective population‐based cohort study was conducted using the Japan Medical Data Center claims database. The overall cohort comprised schizophrenia patients aged ≥ 18 years, who received paliperidone palmitate 1‐month (PP1M) within 180 days before initiating PP3M. Of patients in the overall cohort, those who received PP1M ≥ 4 times within 180 days at 21–42‐day intervals with the same dosage strength as the last two PP1M doses before switching to PP3M initiated PP3M with a dose equivalent to 3.5‐fold the last PP1M dose and took no other concomitant antipsychotics within 112 days before initiating PP3M were included in the per protocol cohort (PPC). The Kaplan–Meier method was used to calculate PP3M persistence rates in the overall cohort and PP3M monotherapy persistence rates in the PPC.

**Results:**

In the overall cohort and PPC, 121 patients and 87 patients, with a mean age of 41.5 years and 48%–53% being employed, were followed up for ≤ 27 months. At 365 days and 730 days, the PP3M persistence rate was 76.9% and 71.7% in the overall cohort, and that for PP3M monotherapy was 73.1% and 64.6% in the PPC.

**Conclusion:**

Treatment persistence rates for PP3M in Japan were relatively high among schizophrenia patients transitioned from PP1M. High persistence rates can be achieved with PP3M monotherapy in patients who have been sufficiently stabilized with PP1M monotherapy prior to initiating PP3M.

## Abstract 2

In Japan, schizophrenia patients need to be stabilized with paliperidone palmitate 1‐month (PP1M) monotherapy prior to switching to paliperidone palmitate 3‐month (PP3M) treatment. However, evidence for the treatment persistence rates of PP3M in real‐world settings is limited. Our results demonstrated high persistence rates for PP3M monotherapy, indicating that such rates can be achieved in patients who have been sufficiently stabilized with PP1M monotherapy before initiating PP3M.

## Graphical abstract

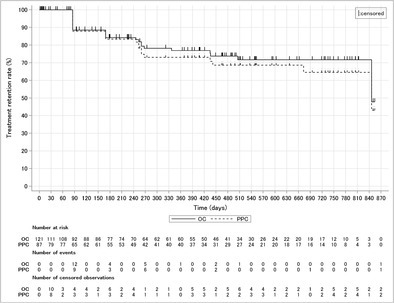

## 1 Introduction

Paliperidone palmitate 3‐month formulation (PP3M) is a long‐acting injectable atypical antipsychotic that has been approved for schizophrenia in Japan since November 2020. The manufacturer's instructions for use in Japan as per the package insert indicate PP3M for the treatment of individuals with schizophrenia who have been stabilized with paliperidone palmitate 1‐month (PP1M) monotherapy within 112 days before switching to PP3M [1]. Several studies from other countries have reported a higher treatment persistence rate and adherence for PP3M than for PP1M in real‐world settings [2, 3, 4]; however, evidence for the use of PP3M in real‐world settings in Japan is limited [5].

Therefore, the study aim was to obtain evidence of PP3M treatment persistence rates by analyzing the claims data of patients who transitioned from PP1M to PP3M in real‐world settings, particularly patients who transitioned according to the Japan‐specific PP3M labeling recommendations.

## 2 Methods

### 2.1 Study Design, Data Source, and Study Cohorts

This was a retrospective population‐based cohort study to examine PP3M treatment persistence rates. We used the Japan Medical Data Center (JMDC) claims database, one of the largest databases in Japan that contains anonymized receipts (inpatient, outpatient, dispensing) and medical examination data received from several insurance associations since 2005 [6, 7]. The database contains data for private sector employees and their dependents and includes data only for individuals aged < 75 years.

Variables of interest generated and analyzed in the study were age at the time of initial diagnosis, sex, and employment status. We extracted claims records from the database to identify patients with a definite diagnosis of schizophrenia (International Classification of Diseases Tenth Revision code F20), aged ≥ 18 years at the time of initial diagnosis, with a record of sex, who received their first PP3M prescriptions between November 2020 and March 2023 and PP1M prescriptions during the period starting 180 days before the first PP3M prescription and ending the day before the first PP3M prescription (overall cohort). Patients who did not receive any PP1M prescription within 180 days before the first PP3M dose were excluded. Among patients in the overall cohort, those who received PP1M ≥ 4 times within 180 days at 21–42‐day dosing intervals before switching to PP3M [1], received the same dosage strength for the last two PP1M treatments as a single dose each, switched to a PP3M dose equivalent to 3.5‐fold the last PP1M dose (patients prescribed with PP1M doses ≤ 39 mg were excluded), and took no concomitant antipsychotics within 112 days before initiating PP3M, were included in the per protocol cohort (PPC).

In an international collaborative study including Japanese patients and a long‐term study in Japanese patients, the subsequent dosing was administered 28 ± 7 days after reaching steady state. In addition, a time profile of plasma paliperidone concentrations showed that a similar concentration could be reattained by resuming treatment at the same dose within 6 weeks after the last dosing. Therefore, the dosing intervals before switching to PP3M were set at 21–42 days [5].

This study was exempt from review by an institutional review board, and informed consent from each patient was not required.

### 2.2 Outcomes

The PP3M treatment persistence rates in the overall cohort and PP3M monotherapy persistence rates in the PPC were evaluated. Patients were classified as an event occurrence if they discontinued the treatment with PP3M. The discontinuation of PP3M treatment was defined as patients who were prescribed PP1M, specifically marking the event as the day of the first PP1M prescription after the initiation of PP3M treatment, or those who were followed up but not prescribed PP3M within 112 days following the last PP3M prescription, marking the event specifically on the 84th day after the last PP3M administration. In addition, patients in the PPC were also classified as an event occurrence if they were prescribed any antipsychotics after the initiation of treatment with PP3M, specifically if the event occurred 84 days after the date of PP3M administration prior to the initiation of the first other antipsychotic.

### 2.3 Analysis

Descriptive analysis was performed to describe the demographics of patients in the overall cohort and PPC, and the data are presented as the number and percentage of patients, or mean ± standard deviation (SD) and range. The Kaplan–Meier method was used to calculate the PP3M persistence rate in the overall cohort and the PP3M monotherapy persistence rate in the PPC. Patients who were lost to follow‐up (e.g., no data as of the data cut‐off date) or completed the study without discontinuing PP3M before March 2023 were treated as censored. All analyses were conducted using SAS version 9.4 (SAS Institute, Tokyo, Japan).

## 3 Results

**FIGURE 1:**
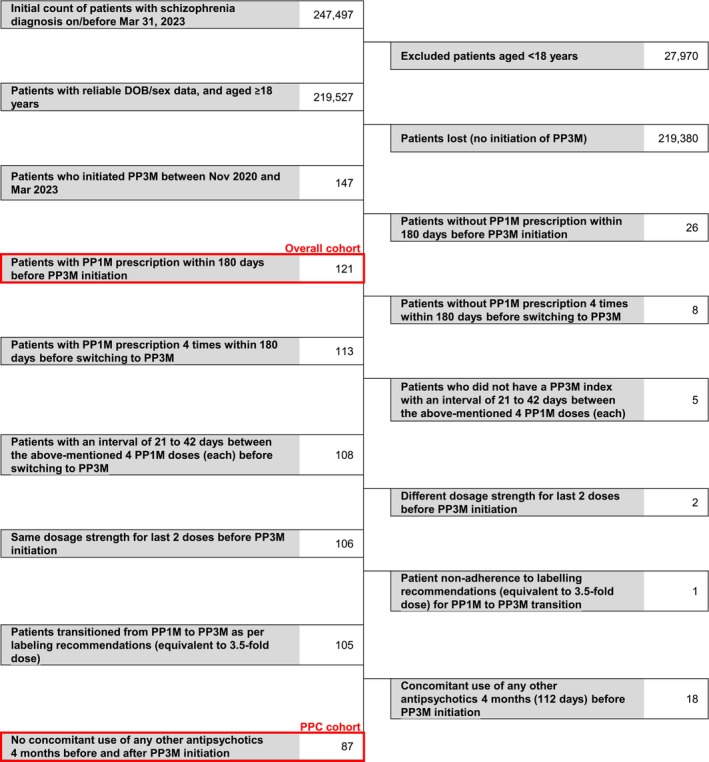
Patient record selection flow. DOB, date of birth; PPC, per protocol cohort; PP1M, paliperidone palmitate 1‐monthly; PP3M, paliperidone palmitate 3‐monthly.

Of 16 572 535 patients registered in the JMDC database as of March 2023, 247 497 patients were diagnosed with schizophrenia on or before March 31, 2023. Of 147 patients who were aged ≥ 18 years and received their first PP3M between November 2020 and March 2023, 121 received a PP1M within 6 months prior to receiving the first PP3M (overall cohort, Figure 1). Furthermore, 87 patients switched from PP1M to PP3M as per the package insert without concomitant antipsychotics other than PP1M within 112 days before switching to PP3M (PPC).

The mean ± SD age was 41.5 ± 9.6 years and 41.2 ± 9.4 years in the overall cohort and PPC, respectively (Table 1). Slightly more women were included (55.4% in the overall cohort and 55.2% in the PPC), and approximately half of the patients were employed (47.9% in the overall cohort and 52.9% in the PPC).

**TABLE 1:** Demographic characteristics of patients.

Variable	Overall cohort (*N* = 121)	PPC (*N* = 87)
Female sex, *n* (%)	67 (55.4)	48 (55.2)
Age, mean (SD), (min, max)	41.50 (9.57) (19.44, 68.74)	41.18 (9.35) (19.44, 68.74)
Employed, *n* (%)	58 (47.9)	46 (52.9)

Abbreviations: PPC, per protocol; SD, standard deviation.

**FIGURE 2:**
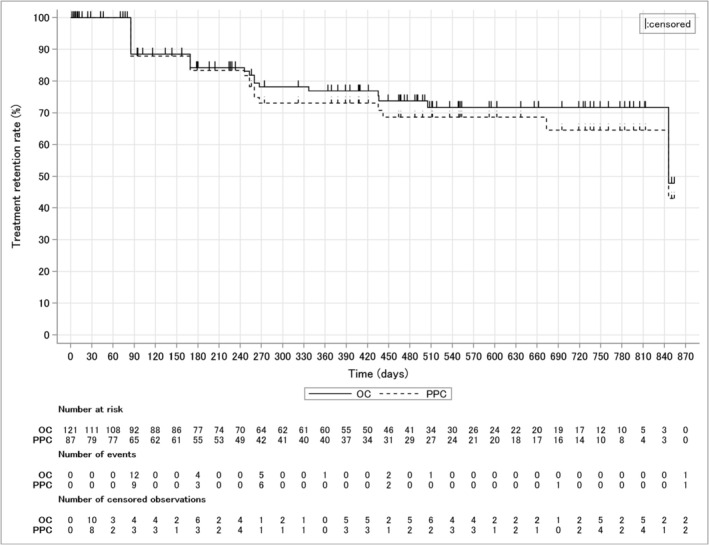
Treatment persistence rate in the overall cohort and PPC. OC, overall cohort; PPC, per protocol cohort.

The treatment persistence rate of PP3M therapy in the overall cohort was 76.9% at 365 days and 71.7% at 730 days (Figure 2). The persistence rate of PP3M in the PPC was 73.1% at 365 days and 64.6% at 730 days.

## 4 Discussion

In the present study, the persistence rate of PP3M in patients who were appropriately switched from PP1M was examined by analyzing the JMDC database claims data.

Instructions on switching treatment from PP1M to PP3M in Japan are stricter than in other countries because patients are required to be treated with PP1M monotherapy within 112 days prior to initiating the PP3M treatment. The present results demonstrate that approximately 72% (87 patients in the PPC out of 121 patients in the overall cohort) of patients were appropriately switched from PP1M to PP3M by following the manufacturer's instructions, indicating that many patients were successfully switched from PP1M to PP3M treatment.

Even after initiating the treatment with PP3M, patients in the PPC were maintained on monotherapy with PP3M. Under such conditions, the persistence rate of monotherapy was 73.1% at 12 months, which is close to that reported in our interim study [5] and by Clark and colleagues [8, 9], suggesting the good treatment persistence of PP3M in real‐world healthcare settings in Japan.

A small proportion of patients (approximately 10%) who initiated with the first dose of PP3M did not transition to the second dose. Notably, the discontinuation rate decreased with each subsequent dose administered. Our findings align with those of another claims database study [3]. The precise reasons for treatment discontinuation remain unclear because most claims databases do not capture detailed information regarding the reason for treatment discontinuation. In the other study based on Taiwan's nationwide claims data analysis [3], it was found that 13.7% of patients who received the first PP3M dose did not transition to the second dose; roughly half of these patients switched back to PP1M, oral paliperidone, or other long‐acting injectable treatments, and it was uncommon for patients to completely discontinue psychiatric services. Nevertheless, the transition from the first to the second dose of PP3M still requires particular attention from clinicians.

In this study, the Kaplan–Meier method was used to present our initial results in a descriptive manner. However, alternative approaches, such as the Cox proportional hazards model, might offer significant advantages in terms of adjusting for confounding factors such as age and sex. Future research will incorporate these analytical methods to enhance the comprehensiveness of the analysis.

In summary, the present results suggest that high persistence rates with PP3M monotherapy can be achieved in patients who have been sufficiently stabilized with PP1M monotherapy prior to initiating PP3M. These results add to the growing body of evidence supporting the effectiveness of PP3M in maintaining treatment persistence under labeling conditions in Japan [10].

## 5 Limitations

There were some study limitations. First, the background characteristics of schizophrenia patients in real‐world settings are likely to be different from those in the database. The database contains data for private sector employees and their dependents and includes data only for individuals aged < 75 years, which might not represent the entire demographic of schizophrenia patients. Approximately 50% of patients in this study were employed, which appeared to be higher than that in real‐world settings. Therefore, this might have impacted the PP3M persistence rate.

Second, the primary objective of this study was to investigate the persistence rates for PP3M under conditions compliant with the Japanese package insert. While concomitant medications, such as psychotropic drugs or treatments for physical conditions, might offer further insights into patients' overall health, this study focused on persistence with PP3M monotherapy. The use of concomitant medications may affect treatment continuation, and future research will explore this to better understand its impact on patient outcomes.

Third, in many countries other than Japan, PP3M can be administered concomitantly with other antipsychotic drugs. The overall cohort was included in this study to allow comparisons with other regions, although PP3M was administered according to the Japanese package insert in some patients. Therefore, the results may not fully reflect practices in other countries. Nonetheless, our findings regarding the persistence rate of PP3M are consistent with studies from Taiwan [3], the U.K. [8, 9], and the U.S.A. [11], suggesting that the overall effectiveness of PP3M remains stable across different populations, despite differences in treatment protocols.

Fourth, patient employment status, which may influence treatment persistence by mediating access and appointment attendance, was not available for analysis in this study. For example, access may not always be easy for working patients because of restrictions related to access times to hospitals and similar facilities. They may also face time constraints that could make attending appointments more challenging. The current study relied on descriptive data because of the limited number of patients using PP3M in this database. These factors are important, and future research will explore them in greater depth once a sufficient number of patients receiving PP3M are available.

Therefore, further studies with larger sample sizes in real‐world clinical settings are needed to validate these findings and to better understand the factors influencing treatment persistence and patient outcomes associated with PP3M.

## 6 Conclusions

Based on the results of the study, in which patients were followed up for up to 27 months, > 70% of patients who were appropriately switched from PP1M continued PP3M for 1 year as monotherapy after the switch. An additional study using a database with a larger sample size, or further analysis using a larger number of cases, is needed before firm conclusions can be drawn about the PP3M treatment persistence rate in real‐world clinical settings in Japan.

## Author Contributions

A.W., M.C., H.H., and Y.S. designed the study. A.W. and H.H. helped interpret the results and assisted with the writing of the manuscript. C.‐L.C. supervised the project. A.W., H.H., and N.T. wrote the manuscript with support from Y.S. All authors discussed the results and commented on the manuscript.

## Consent

The authors have nothing to report.

## Conflicts of Interest

Chih‐Lin Chiang is an employee of Janssen Pharmaceutical, Johnson & Johnson, Taiwan. All other authors are employees of Janssen Pharmaceutical K.K., Japan.

## Data Availability

The data sharing policy of Janssen Pharmaceutical Companies of Johnson & Johnson is available at https://www.janssen.com/clinical‐trials/transparency. These data were made available by JMDC Inc. and used under license for the current study and are not publicly available. Other researchers should contact their website at https://www.jmdc.co.jp/en/.
